# Supplementary material for: hGRAD: A versatile “one-fits-all” system to acutely deplete RNA binding proteins from condensates
Source: J Cell Biol. 2023 Dec 18;223(2):e202304030. doi: 10.1083/jcb.202304030 (PMC10726014; doi:10.1083/jcb.202304030)
Supplement: Table S2 — provides the list of gRNAs. [file JCB_202304030_TableS2.docx]

**Table S2: List of guide RNAs (gRNAs).** crRNA - CRISPR RNA.

| Species | Name/Target | Protospacer sequence | PAM | Purpose | Supplier |
| --- | --- | --- | --- | --- | --- |
| Human | SRSF3 crRNA | gtcgatctaggtcaaatgaa | AGG | HDR, C-term. GFP-tag | IDT |
| Human | SRSF5 crRNA | aggtccagatcagttgacag | TGG | HDR, C-term. GFP-tag | IDT |
| Human | SRRM2 crRNA | ttccaccacacccaatgctc | TGG | HDR, C-term. GFP-tag | IDT |
| Mouse | SRSF3 crRNA | gccgatctaggtcaaatgaa | AGG | HDR, C-term. GFP-tag | IDT |
| Mouse | SRSF5 crRNA | aggtccagatcagttgacag | TGG | HDR, C-term. GFP-tag | IDT |
| - | universal tracrRNA | - | - | - | IDT |
