# Supplementary material for: hGRAD: A versatile “one-fits-all” system to acutely deplete RNA binding proteins from condensates
Source: J Cell Biol. 2023 Dec 18;223(2):e202304030. doi: 10.1083/jcb.202304030 (PMC10726014; doi:10.1083/jcb.202304030)
Supplement: Table S3 — lists antibodies used in this study. [file JCB_202304030_TableS3.docx]

**Table S3: List of antibodies used in this study.**

| Name | Species | Supplier | Catalog-Nr. |
| --- | --- | --- | --- |
| α-Camelid-VHH | rabbit | Genscript | A01860 |
| α-GFP | goat | Eric Geertsma, MPI-CBG | - |
| α-mCherry | rabbit | Abcam | ab167453 |
| α-SRp40 (SRSF5) | rabbit | Merck Millipore | 06-1365 |
| α-goat-HRP | donkey | Sigma Aldrich | AB324P |
| α-rabbit-HRP | donkey | Merck Millipore | AP182P |
| α-rabbit-Alexa Fluor 680 | donkey | Thermo Fisher Scientific | A10043 |
| α-rabbit-Alexa Fluor Plus 405 | donkey | Thermo Fisher Scientific | A48258 |
| α-SRRM2 | rabbit | Thermo Fisher Scientific | PA5-59559 |
