# Supplementary material for: hGRAD: A versatile “one-fits-all” system to acutely deplete RNA binding proteins from condensates
Source: J Cell Biol. 2023 Dec 18;223(2):e202304030. doi: 10.1083/jcb.202304030 (PMC10726014; doi:10.1083/jcb.202304030)
Supplement: Table S4 — lists the plasmids used or generated in this study. [file JCB_202304030_TableS4.docx]

**Table S4: List of plasmids used or generated in this study.**

| Name | Vendor | Catalog Nr. | Reference |
| --- | --- | --- | --- |
| pmCherry-N1 | Clontech | 632523 | - |
| pGEM®-T Easy | Promega | A1360 | - |
| pTRE-BI | VectorBuilder | VB190904-1039fwc | This work |
| pSH-EFIRES-P-AtAFB2-mCherry-weak NLS | Addgene | 129717 | (Li et al., 2019) |
| pUAST_NSlmb-vhhGFP4 | Addgene | 35575 | (Caussinus et al., 2011) |
| pCAGGs-NLS-TIR1_P2A_NES-TIR1 | Addgene | 117699 | (Daniel et al., 2018) |
| pIRES_IBB-mCherry-IRES-HA-mAID-nanobody | Addgene | 117720 | (Li et al., 2019) |
| pSH-EFIRES-P-Seipin-miniIAA7-3XFlag | Addgene | 129722 | (Li et al., 2019) |
| pFUSE-hIgG1-Fc2 | Invivogen | #pfuse-hg1fc2 | - |
| pTRE-BI-hGRAD-mCherry | - | - | This work |
| pTRE-BI-TRIM21-mCherry-hIgG1FC2 | - | - | This work |
| pTRE-BI-TIR1-mCherry-mAID | - | - | This work |
| pTRE-BI-AFB2-mCherry-miniIAA7 | - | - | This work |
| pGEMT-HDR-hSRSF3-GFP | - | - | This work |
| pGEMT-HDR-mSRSF3-GFP | - | - | This work |
| pGEMT-HDR-hSRSF5-GFP | - | - | This work |
| pGEMT-HDR-mSRSF5-GFP | - | - | This work |
| pGEMT-HDR-hSRRM2-GFP | - | - | This work |
