# Supplementary material for: hGRAD: A versatile “one-fits-all” system to acutely deplete RNA binding proteins from condensates
Source: J Cell Biol. 2023 Dec 18;223(2):e202304030. doi: 10.1083/jcb.202304030 (PMC10726014; doi:10.1083/jcb.202304030)
Supplement: Table S5 — lists BACs used in this study. [file JCB_202304030_TableS5.docx]

**Table S5: List of BACs used in this study.**

| Internal-Nr. | Name | Selection marker |
| --- | --- | --- |
| MCB#2951 | mSRSF5-GFP | Geneticin / Chloramphenicol |
| MCB#6553 | hSRSF5-GFP | Geneticin / Chloramphenicol |
| MCB#5415 | mSRSF3-GFP | Geneticin / Chloramphenicol |
| MCB#3204 | mSRPK1-GFP | Geneticin / Chloramphenicol |
| MCB#5404 | mNONO-GFP | Geneticin / Chloramphenicol |
| MCB#8367 | mEIF4E-GFP | Geneticin / Chloramphenicol |
