# Supplementary material for: hGRAD: A versatile “one-fits-all” system to acutely deplete RNA binding proteins from condensates
Source: J Cell Biol. 2023 Dec 18;223(2):e202304030. doi: 10.1083/jcb.202304030 (PMC10726014; doi:10.1083/jcb.202304030)
Supplement: Table S6 — list of cell lines used or generated in this study (selection marker: puromycin [Puro] or geneticin [Gen] as indicated). [file JCB_202304030_TableS6.docx]

**Table S6: List of cell lines used or generated in this study (selection marker: puromycin (Puro) or geneticin (Gen) as indicated).**

| Name | Selection marker | Source | Species |
| --- | --- | --- | --- |
| HeLa wild type | - | ATTC^®^ | Human |
| HeLa hGRAD | Puro | This work | Human |
| HeLa TRIM21 | Puro | This work | Human |
| HeLa TIR1 | Puro | This work | Human |
| HeLa AFB2 | Puro | This work | Human |
| HeLa hGRAD SRSF3-GFP (BAC) | Puro / Gen | This work | Human |
| HeLa TRIM21 SRSF3-GFP (BAC) | Puro / Gen | This work | Human |
| HeLa TIR1 SRSF3-GFP (BAC) | Puro / Gen | This work | Human |
| HeLa AFB2 SRSF3-GFP (BAC) | Puro / Gen | This work | Human |
| HeLa hGRAD SRSF5-GFP (BAC) | Puro / Gen | This work | Human |
| HeLa hGRAD SRPK1-GFP (BAC) | Puro / Gen | This work | Human |
| HeLa hGRAD EIF4E-GFP (BAC) | Puro / Gen | This work | Human |
| HeLa hGRAD NONO-GFP (BAC) | Puro / Gen | This work | Human |
| HeLa hGRAD SRSF3-GFP (HDR) | Puro / Gen | This work | Human |
| HeLa hGRAD SRSF5-GFP (HDR) | Puro / Gen | This work | Human |
| HeLa hGRAD SRRM2-GFP (HDR) | Puro / Gen | This work | Human |
| P19 wild type | - | Sigma Aldrich / ECACC | Mouse |
| P19 hGRAD | Puro | This work | Mouse |
| P19 hGRAD SRSF3-GFP (BAC) | Puro / Gen | This work | Mouse |
| P19 hGRAD SRSF5-GFP (BAC) | Puro / Gen | This work | Mouse |
| P19 hGRAD SRPK1-GFP (BAC) | Puro / Gen | This work | Mouse |
| P19 hGRAD EIF4E-GFP (BAC) | Puro / Gen | This work | Mouse |
| P19 hGRAD SRSF3-GFP (HDR) | Puro / Gen | This work | Mouse |
| P19 hGRAD SRSF5-GFP (HDR) | Puro / Gen | This work | Mouse |
