# Supplementary material for: hGRAD: A versatile “one-fits-all” system to acutely deplete RNA binding proteins from condensates
Source: J Cell Biol. 2023 Dec 18;223(2):e202304030. doi: 10.1083/jcb.202304030 (PMC10726014; doi:10.1083/jcb.202304030)
Supplement: Table S7 — provides the list of primers used in this study. [file JCB_202304030_TableS7.docx]

**Table S7: List of primers used in this study.**

Primers for cloning of rapid depletion vectors

| **Primer** | **Primer sequence (5'-3')** | **Insert / Amplicon** |
| --- | --- | --- |
| GBA Bi hGRADE ins1 f | ctttcttgtacaaagtgggctagcatggagcccgactcg | Insert 1 hFBXW11 |
| GBA Bi hGRADE ins1 r | agttggacgtgtcgtccaca | Insert 1 hFBXW11 |
| GBA Bi hGRADE ins2 f | acgacacgtccaactggtggagtctgg | Insert 2 VHH-GFP4 |
| GBA Bi hGRADE ins2 r v2 | aactagaaggcacaggatatcttagctggagacggtgacc | Insert 2 VHH-GFP4 |
| mCherry BamHi f | atatGGATCCTCGCCACCATGGTGAG | mCherry |
| mCherry SpeI r | atatACTAGTCTACTTGTACAGCTCGTCC | mCherry |
| GBA Bi AFB2 ins1 f | tggtaccgtcgacggatccctaatcaagtttgacccgtttggc | Insert 1 AtAFB2-mCherry-weak NLS |
| GBA Bi AFB2 ins1 r | ctcgtgatcgatgaactactttcccgatgagg | Insert 1 AtAFB2-mCherry-weak NLS |
| GBA Bi AFB2 ins2 f | gtagttcatcgatcacgagactagcctcg | Insert 2 pTRE-BI promoter |
| GBA Bi AFB2 ins2 r | gaagcccataattcggggccgcg | Insert 2 pTRE-BI promoter |
| GBA Bi AFB2 ins3 f | cccgaattatgggcttctctgagaccg | Insert 3 miniIAA7 |
| GBA Bi AFB2 ins3 r | caccgatatccttgtcatcgtcatccttgtaatcga | Insert 3 miniIAA7 |
| GBA Bi AFB2 ins4 f | gatgacaaggatatcggtgcaggcgg | Insert 4 VHH-GFP4 |
| GBA Bi AFB2 ins4 r | aggatatctgcagaattcgctagcttagctggagacggtgacct | Insert 4 VHH-GFP4 |
| GBA Bi TIR1 ins1 f | tggtaccgtcgacggatccctaatcaagtttgacccgtttggc | Insert 1 mCherry-weak NLS |
| GBA Bi TIR1 ins1 r | aaaatactgggcggatctggc | Insert 1 mCherry-weak NLS |
| GBA Bi TIR1 ins2 f | gatccgcccagtattttcacgaagtttggagcg | Insert 2 TIR1 |
| GBA Bi TIR1 ins2 r | tcgtgatcgatgacgtacttccctgaagaagt | Insert 2 TIR1 |
| GBA Bi TIR1 ins3 f | tacgtcatcgatcacgagactagcctcg | Insert 3 pTRE-BI promoter |
| GBA Bi TIR1 ins3 r | gtaggccataattcggggccgcg | Insert 3 pTRE-BI promoter |
| GBA Bi TIR1 ins4 f | cccgaattatggcctacccatacgatgt | Insert 4 mAID-VHH-GFP4 |
| GBA Bi TIR1 ins4 r | aggatatctgcagaattcgctagcttagctggagacggtgacct | Insert 4 mAID-VHH-GFP4 |
| GBA Bi TRIM21 ins1 f | tggtaccgtcgacggatccctaatcaagtttgacccgtttggc | Insert 1 mCherry-weak NLS |
| GBA Bi TRIM21 ins1 r | actgactatggcggatctggcgg | Insert 1 mCherry-weak NLS |
| GBA Bi TRIM21 ins2 f | gatccgccatagtcagtggatccttgtgatccaatatt | Insert 2 TRIM21 |
| GBA Bi TRIM21 ins2 r | cgtgatcgATGgcttcagcagcacg | Insert 2 TRIM21 |
| GBA Bi TRIM21 ins3 f | gaagcCATcgatcacgagactagcctcg | Insert 3 pTRE-BI promoter |
| GBA Bi TRIM21 ins3 r | ATCTAACCATaattcggggccgcg | Insert 3 pTRE-BI promoter |
| GBA Bi TRIM21 ins4 f | ccccgaattATGGTTAGATCTGACAAAACTCACACA | Insert 4 hIgG1 FC2 |
| GBA Bi TRIM21 ins4 r | accgatatcTTTACCCGGAGACAGGGAGAG | Insert 4 hIgG1 FC2 |
| GBA Bi TRIM21 ins5 f | CCGGGTAAAgatatcggtgcaggcgg | Insert 5 VHH-GFP4 |
| GBA Bi TRIM21 ins5 r | aggatatctgcagaattcgctagcttagctggagacggtgacct | Insert 5 VHH-GFP4 |

Primers for cloning of HDR templates

| Primer | Primer sequence (5'-3') | Insert / Amplicon |
| --- | --- | --- |
| HDR hSRSF3-GFP Ins1 F | cgcgggaattcgattggacgatgggtgcc | Insert 1 5'-HA |
| HDR hSRSF3-GFP Ins1 R | ggaatatcataatcttttctttcatttgacctagatcgactaaaaaaaaaaaaaaaaaaagaa | Insert 1 5'-HA |
| HDR hSRSF3-GFP Ins2 F | aaatgaaagaaaagattatgatattccaactactgcaagcg | Insert 2 GFP-IRES-NeoR |
| HDR hSRSF3-GFP Ins2 R | caaactgtctttcagaagaactcgtcaagaaggc | Insert 2 GFP-IRES-NeoR |
| HDR hSRSF3-GFP Ins3 F | gttcttctgaaagacagtttgcaagagaagtgg | Insert 3 3'-HA |
| HDR hSRSF3-GFP Ins3 R | cgcgaattcactagtgattgaatcgtgtaaccaactgcttcc | Insert 3 3'-HA |
| HDR mSRSF3-GFP Ins1 F | cgcgggaattcgattggtttgcatgggttccaaatact | Insert 1 5'-HA |
| HDR mSRSF3-GFP Ins1 R | ggaatatcataatcttttctttcatttgacctagatcggct | Insert 1 5'-HA |
| HDR mSRSF3-GFP Ins2 F | aaatgaaagaaaagattatgatattccaactactgcaagcg | Insert 2 GFP-IRES-NeoR |
| HDR mSRSF3-GFP Ins2 R | aactggtctttcagaagaactcgtcaagaaggc | Insert 2 GFP-IRES-NeoR |
| HDR mSRSF3-GFP Ins3 F | gttcttctgaaagaccagtttgcaaaagtgg | Insert 3 3'-HA |
| HDR mSRSF3-GFP Ins3 R | cgcgaattcactagtgattcctgaactggcttcaacac | Insert 3 3'-HA |
| HDR hSRSF5-GFP Ins1 F | cgcgggaattcgattggatctcaaagatttcatgagacaagc | Insert 1 5'-HA |
| HDR hSRSF5-GFP Ins1 R | tatcataatcattactgtcaactgatctggacct | Insert 1 5'-HA |
| HDR hSRSF5-GFP Ins2 F | tcagttgacagtaatgattatgatattccaactactgcaagcG | Insert 2 GFP-IRES-NeoR |
| HDR hSRSF5-GFP Ins2 R | gttatttacagttcagaagaactcgtcaagaaggc | Insert 2 GFP-IRES-NeoR |
| HDR hSRSF5-GFP Ins3 F | gagttcttctgaactgtaaataacttgccctggg | Insert 3 3'-HA |
| HDR hSRSF5-GFP Ins3 R | cgcgaattcactagtgattggctgaggcaggagaatcg | Insert 3 3'-HA |
| HDR mSRSF5-GFP Ins1 F | cgcgggaattcgattgtgtggtatatgccatgtttgtactt | Insert 1 5'-HA |
| HDR mSRSF5-GFP Ins1 R | tatcataatcattactgtcaactgatctggacctg | Insert 1 5'-HA |
| HDR mSRSF5-GFP Ins2 F | tcagttgacagtaatgattatgatattccaactactgcaagcG | Insert 2 GFP-IRES-NeoR |
| HDR mSRSF5-GFP Ins2 R | gttatttacagttcagaagaactcgtcaagaaggc | Insert 2 GFP-IRES-NeoR |
| HDR mSRSF5-GFP Ins3 F | gagttcttctgaactgtaaataacttgccctggg | Insert 3 3'-HA |
| HDR mSRSF5-GFP Ins3 R | cgcgaattcactagtgattccctgttctacaaattgagttccaggact | Insert 3 3'-HA |
| HDR hSRRM2-GFP Ins1 F | cgcgggaattcgattcacccagcgccttgc | Insert 1 5'-HA |
| HDR hSRRM2-GFP Ins1 R | atatcataatctggagacctgcaagagaagatatg | Insert 1 5'-HA |
| HDR hSRRM2-GFP Ins2 F | caggtctccagattatgatattccaactactgcaagcG | Insert 2 GFP-IRES-NeoR |
| HDR hSRRM2-GFP Ins2 R | gtggctccatcagaagaactcgtcaagaaggc | Insert 2 GFP-IRES-NeoR |
| HDR hSRRM2-GFP Ins3 F | tcttctgatggagccactgtcccttcttccccagcag | Insert 3 3'-HA |
| HDR hSRRM2-GFP Ins3 R | cgcgaattcactagtgattgggtcccactccctg | Insert 3 3'-HA |

Primers for qRT-PCR

| Primer | Primer Sequence (5'-3') | Insert / Amplicon |
| --- | --- | --- |
| U6_F | gctcgcttcggcagc | *U6* snRNA qPCR Normalization |
| U6_R | aaatatggaacgcttcacgaatt | *U6* snRNA qPCR Normalization |
| hSRSF5_F | ctcactttgagggcaagcct | *SRSF5* |
| hSRSF5_R | ccggctagtacttcctcgaat | *SRSF5* |
| ENSG00000231412_F | atcgcatcccctgtgacttg | *ENSG00000231412* |
| ENSG00000231412_R | ggagcttctgagccaggaaa | *ENSG00000231412* |
| ENSG00000259621_F | gcactcattgttctcggtgc | *ENSG00000259621* |
| ENSG00000259621_R | tgagtgatctggtgctggtg | *ENSG00000259621* |
