# Supplementary figures and images for: hGRAD: A versatile “one-fits-all” system to acutely deplete RNA binding proteins from condensates
Source: J Cell Biol. 2023 Dec 18;223(2):e202304030. doi: 10.1083/jcb.202304030 (PMC10726014; doi:10.1083/jcb.202304030)

FIG.2B

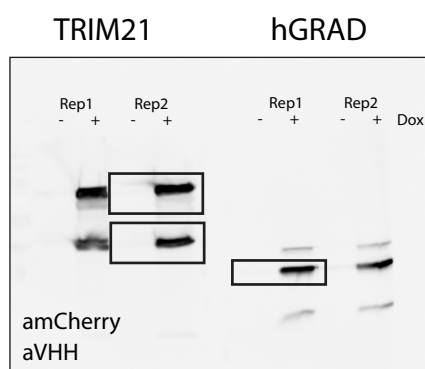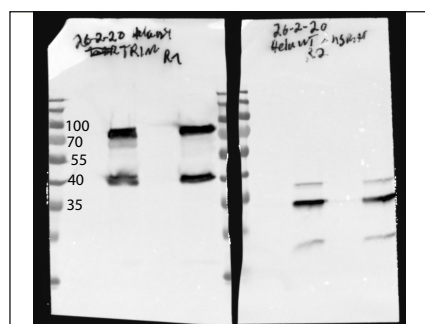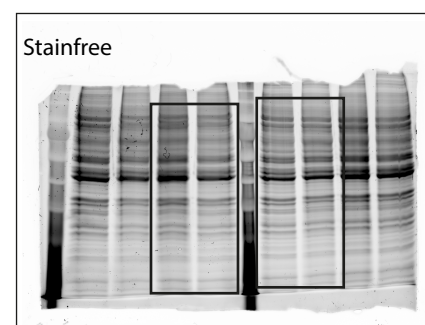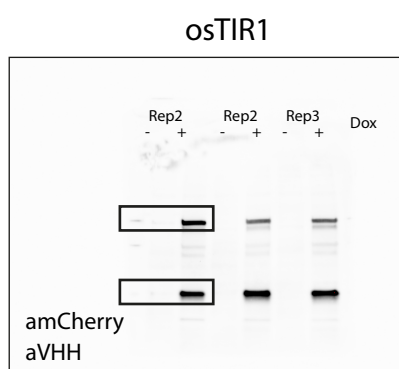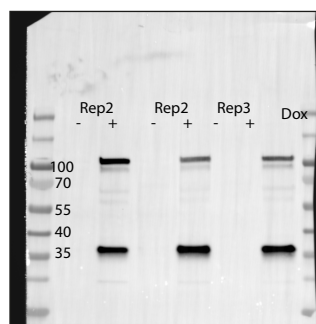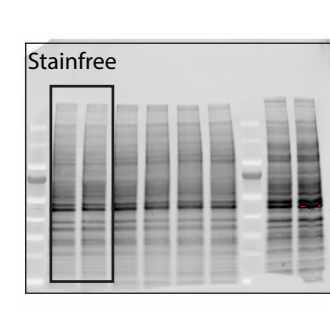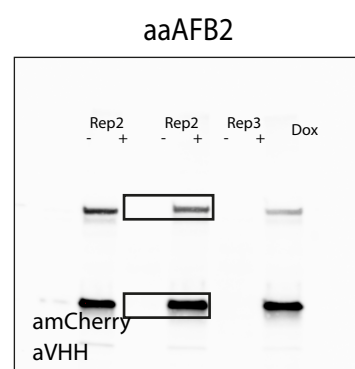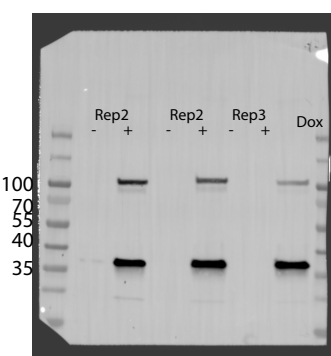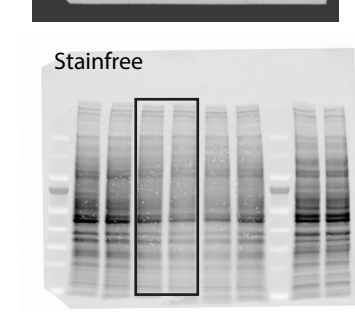

FIG 2D

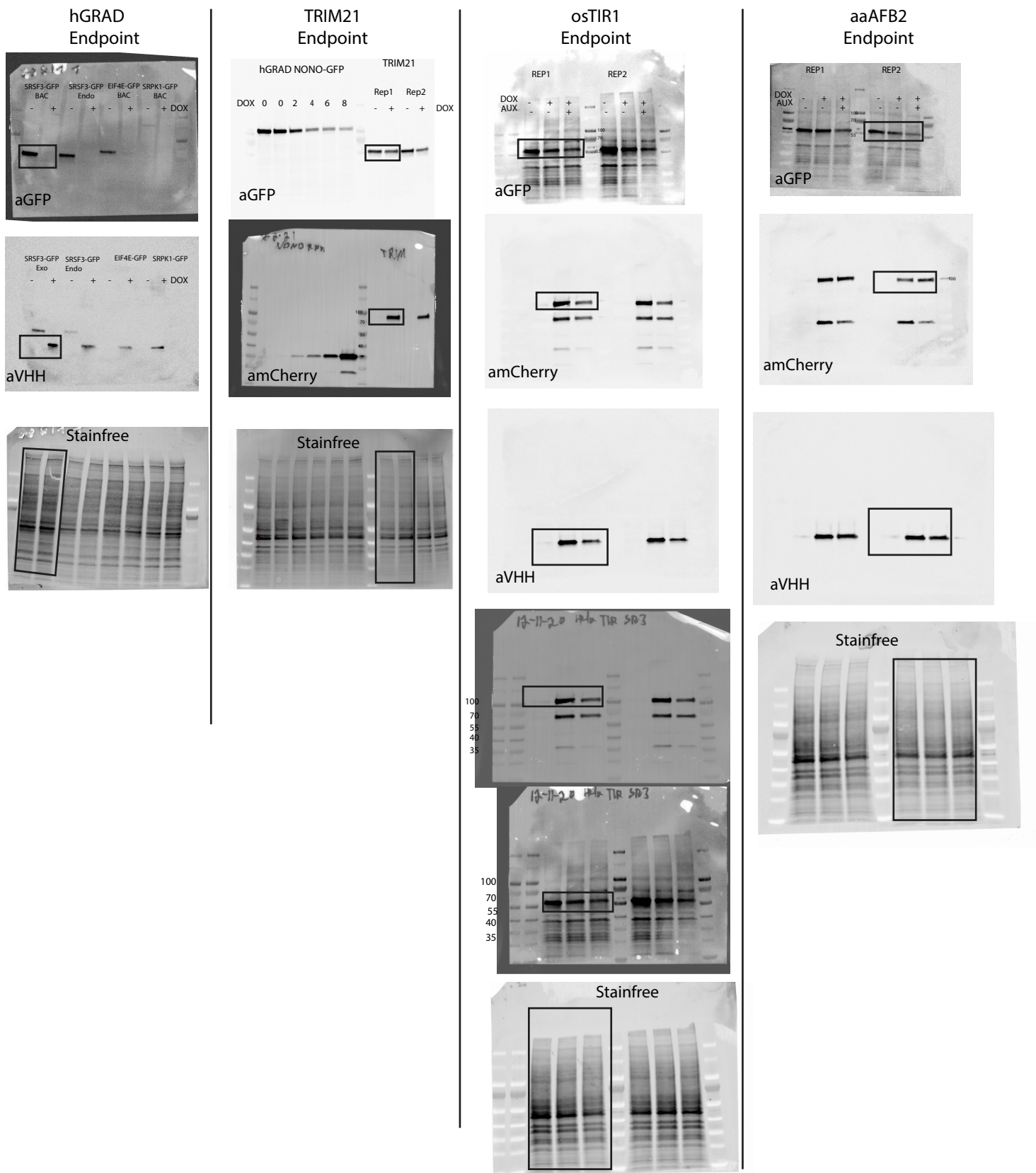

FIG 2E

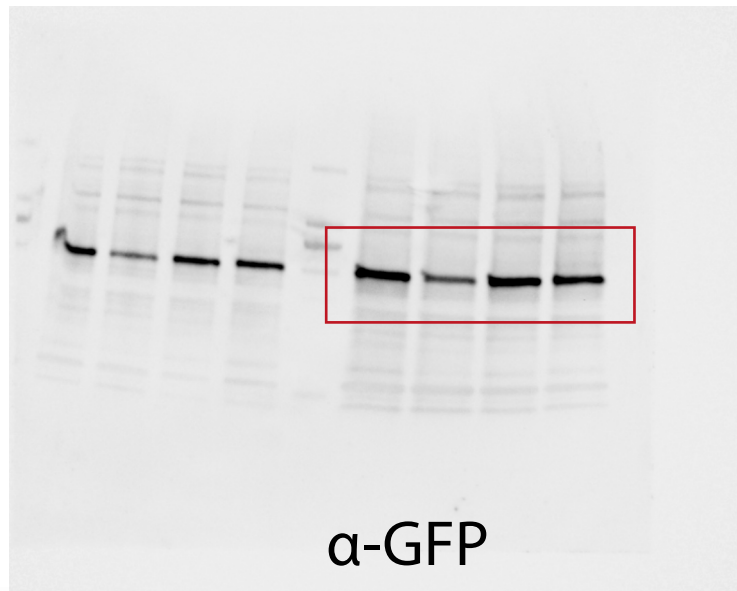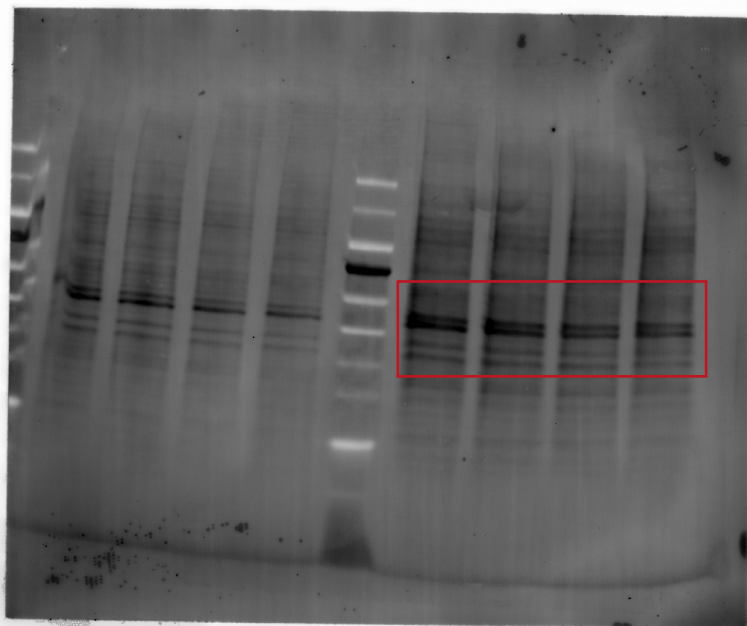

Stain free membrane

Supplement: SourceData F2 — is the source file for Fig. 2. [file JCB_202304030_SourceDataF2.pdf]

Fig 3 B

HeLa hGRAD + BACs

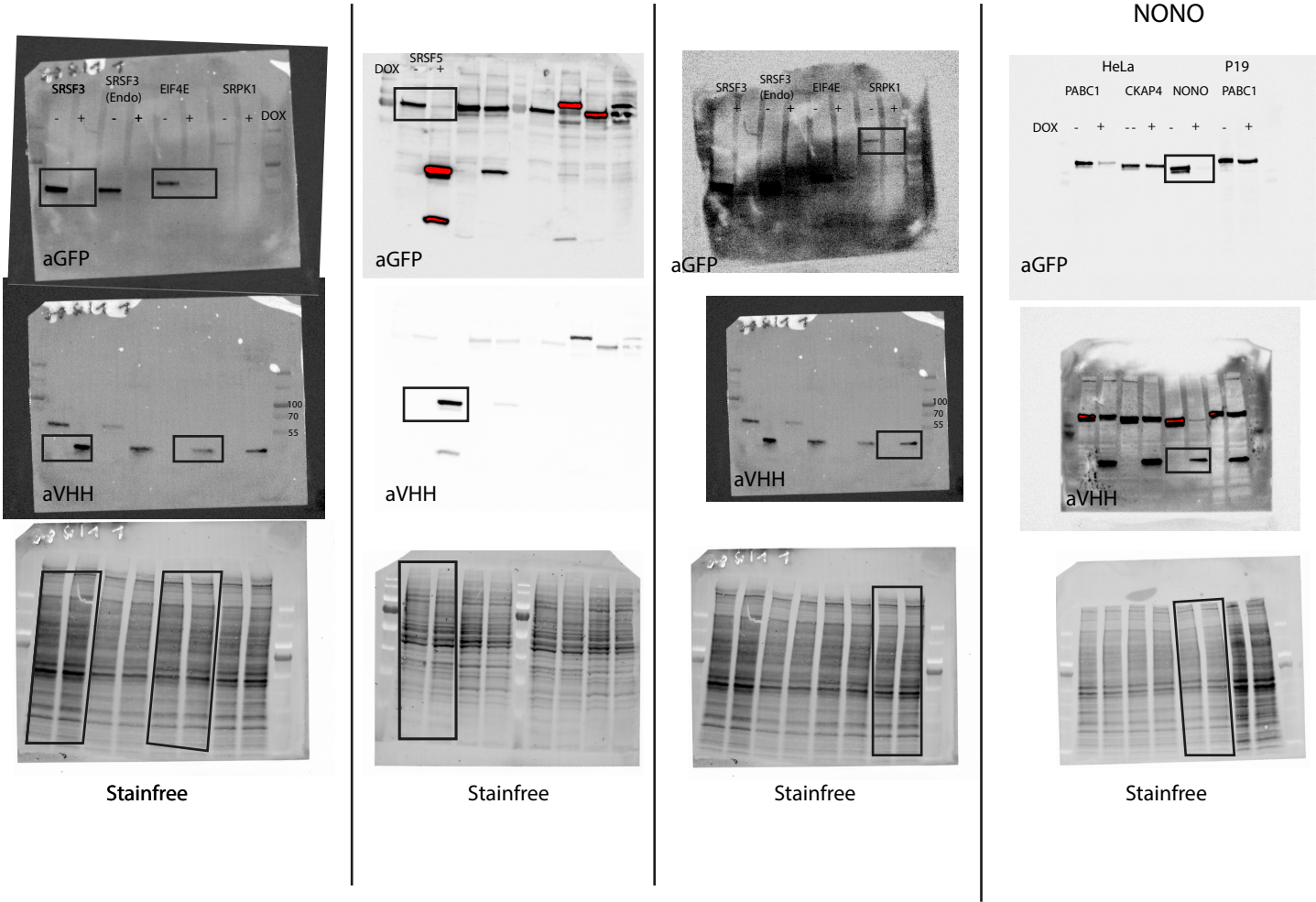

FIG.3C

HeLa hGRAD + BACs

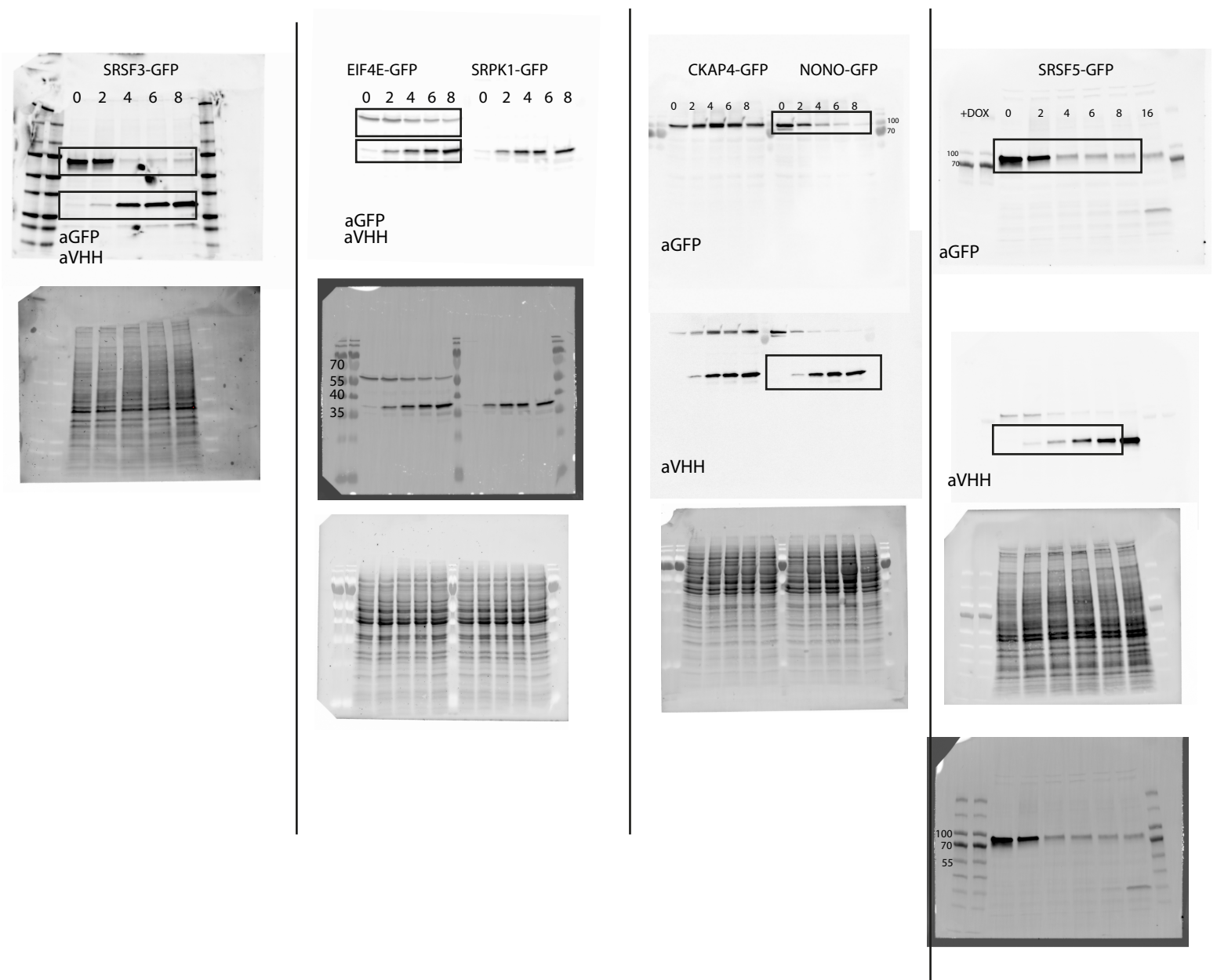

Supplement: SourceData F3 — is the source file for Fig. 3. [file JCB_202304030_SourceDataF3.pdf]

Fig. 5B

HeLa hGRAD endo-GFP

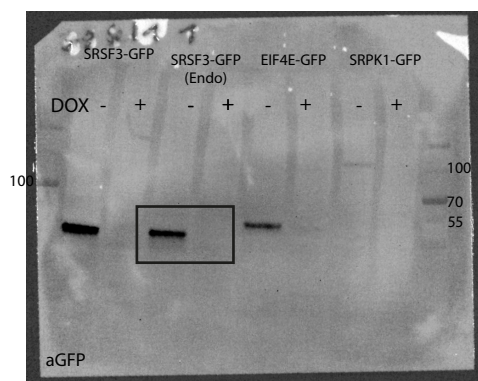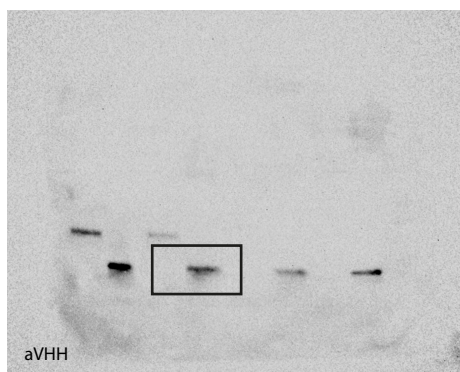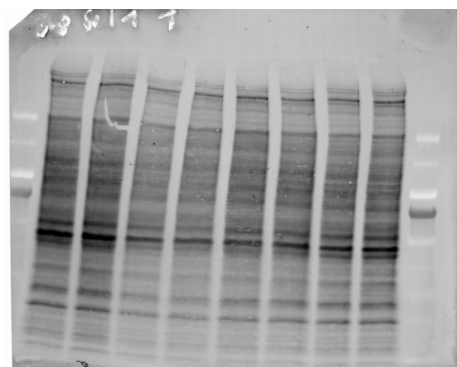

Stainfree

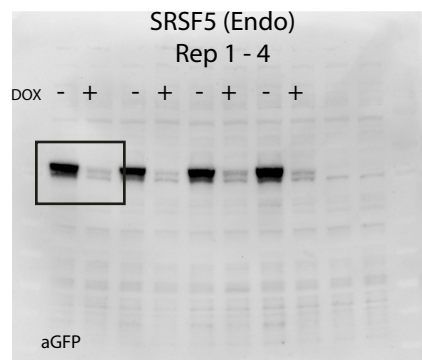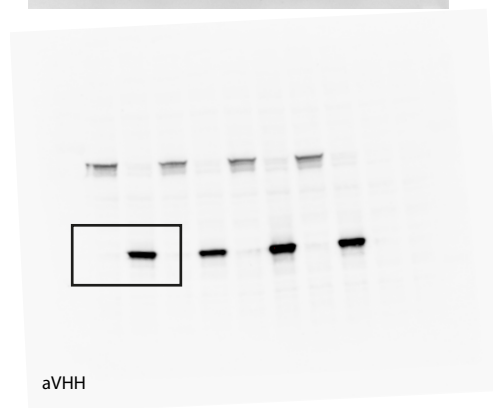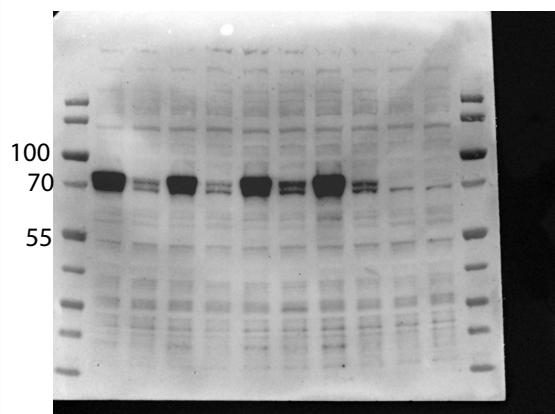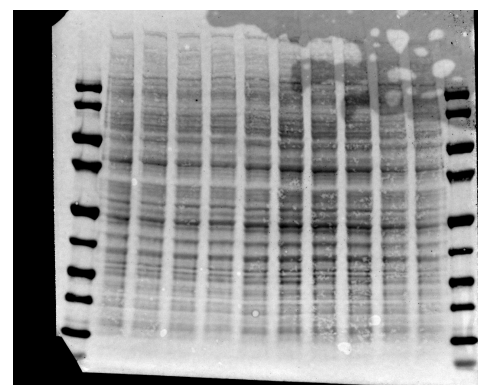

Stainfree

Fig.5C

HeLa hGRAD endo-GFP  
(Timeline)

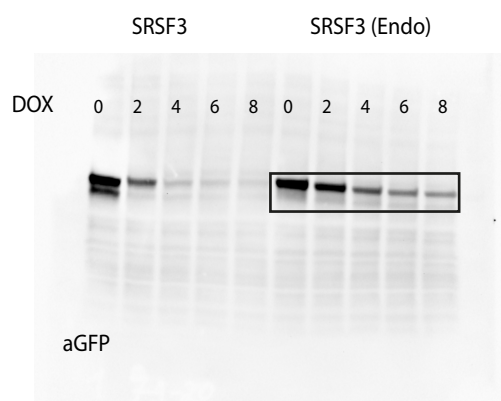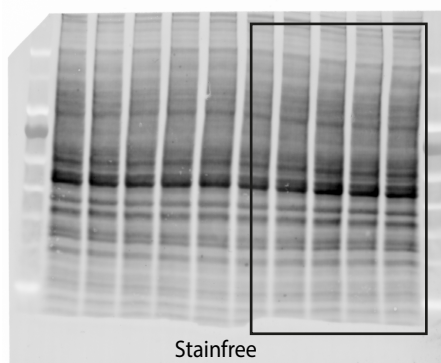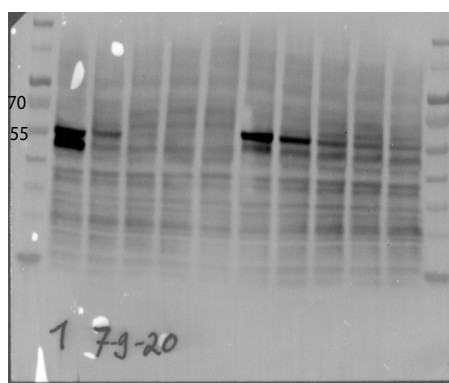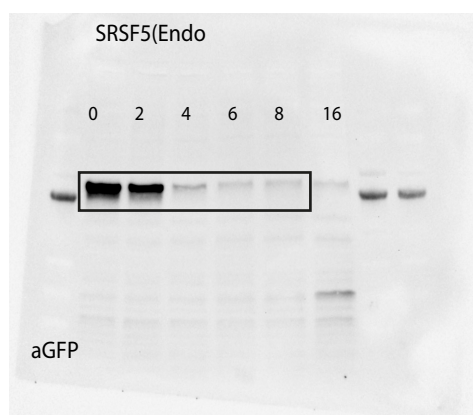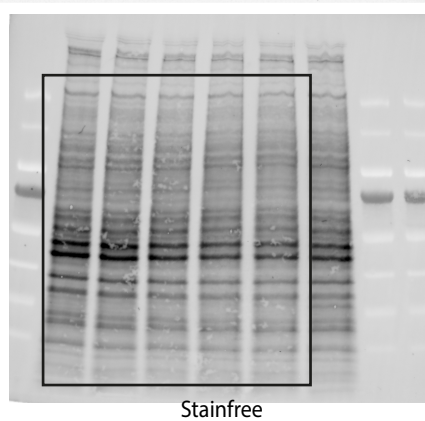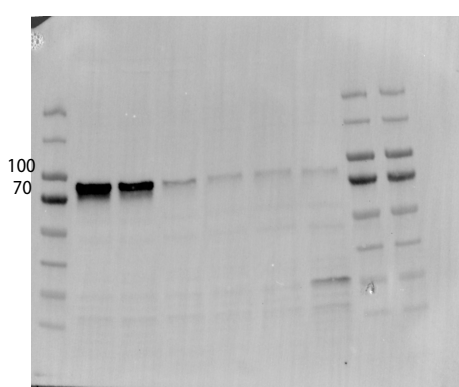

SRRM2-HDR

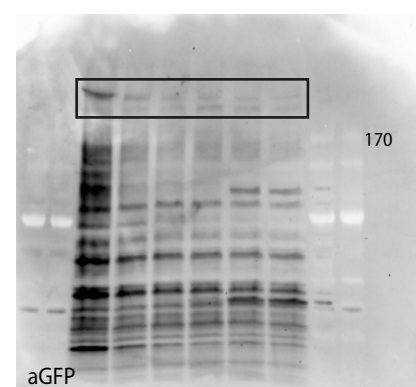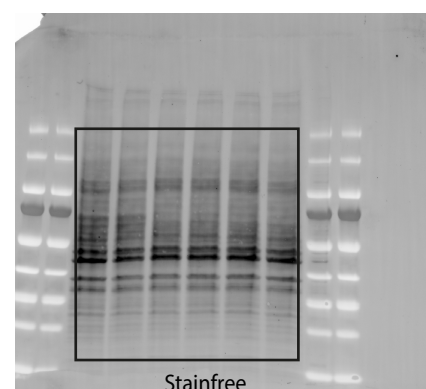

Supplement: SourceData F5 — is the source file for Fig. 5. [file JCB_202304030_SourceDataF5.pdf]

**S3B**  
SRSF5-endo-GFP KI  
HeLa

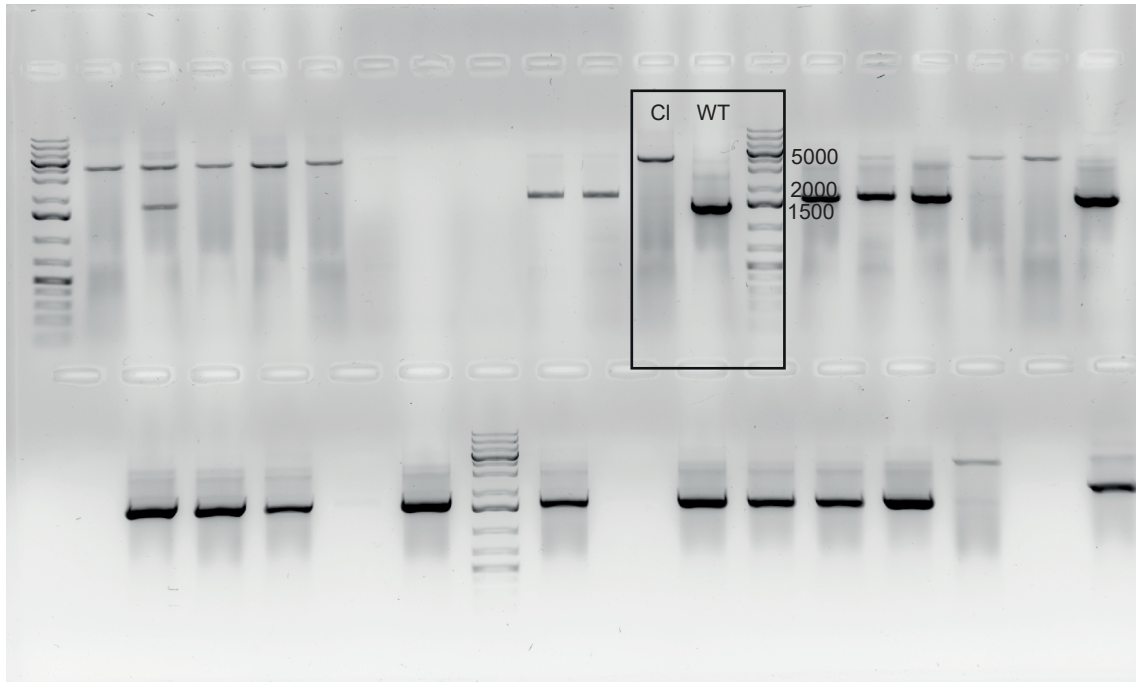

SRSF5-endo-GFP KI  
P19

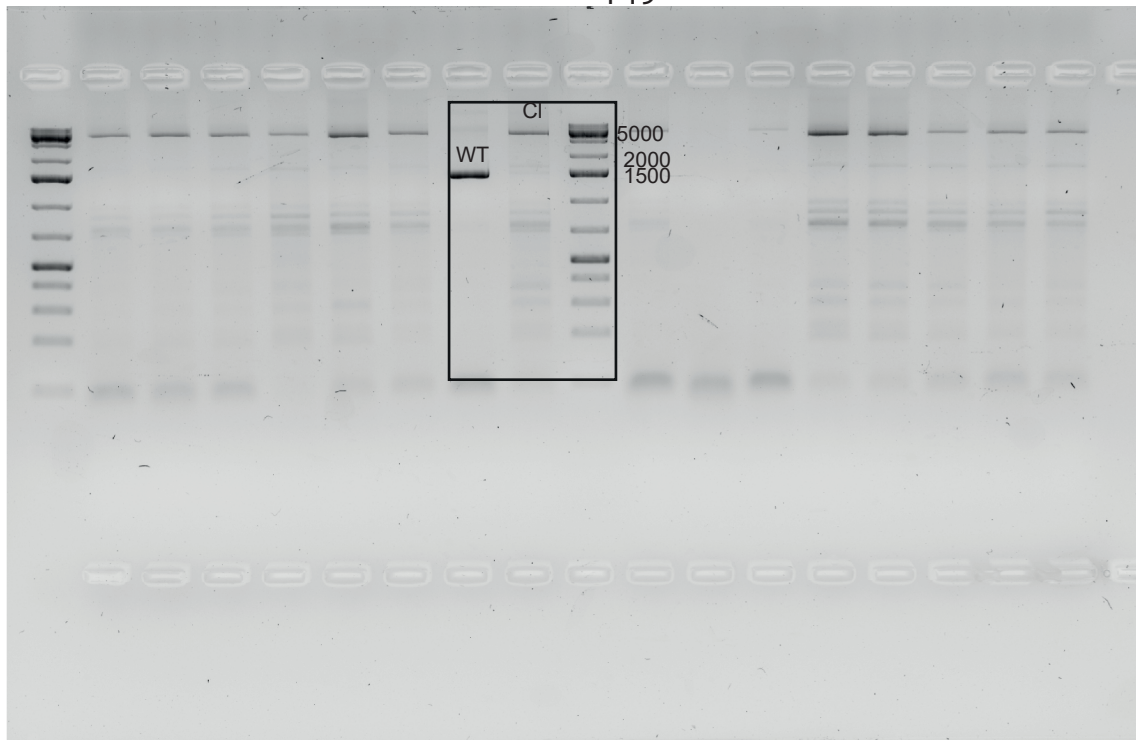

S3C

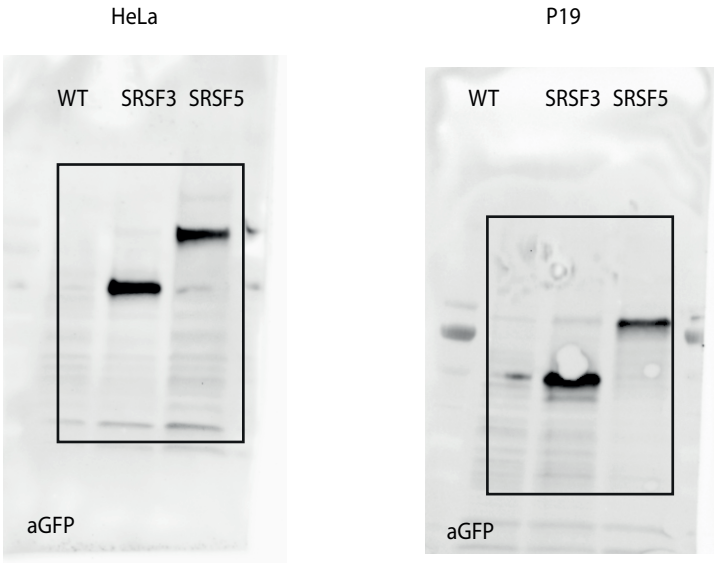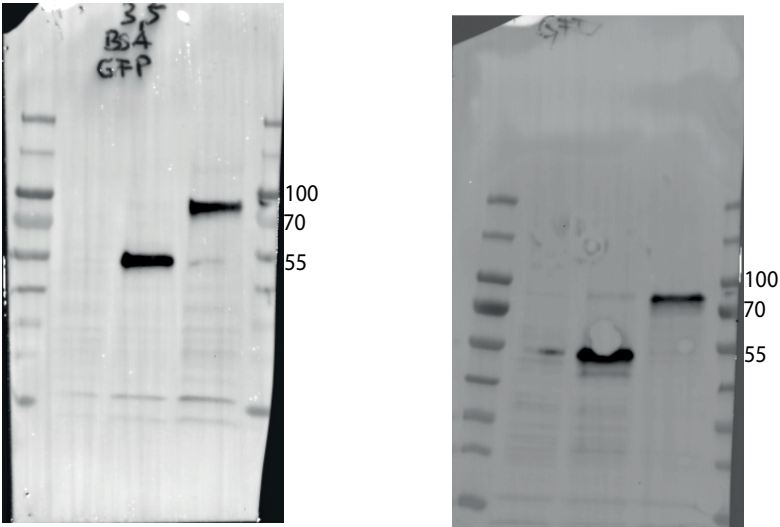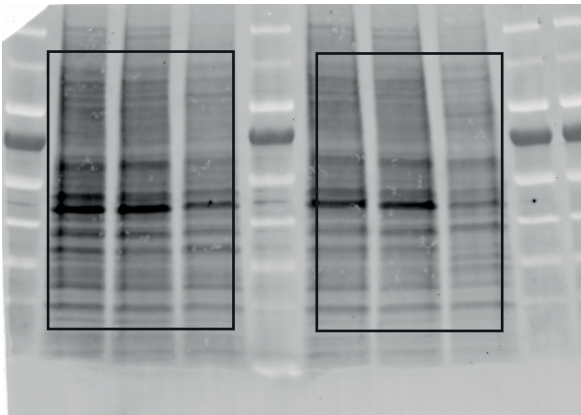

Stainfree

Fig.3SD  
HeLa SRSF5-endo GFP

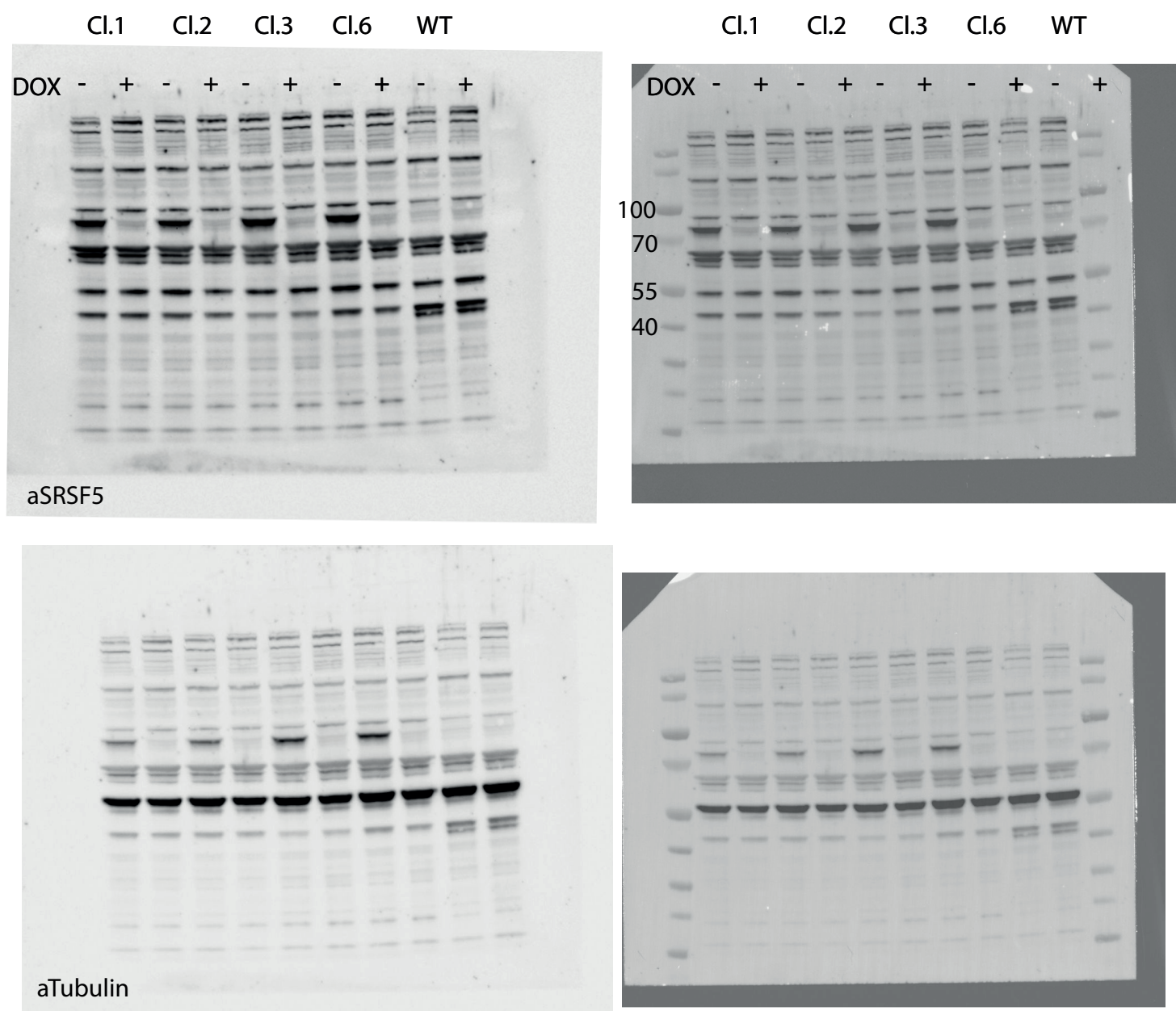

Supplement: SourceData FS3 — is the source file for Fig. S3. [file JCB_202304030_SourceDataFS3.pdf]

S4A

P19 endo-GFP

P19 SRSF3 Endo

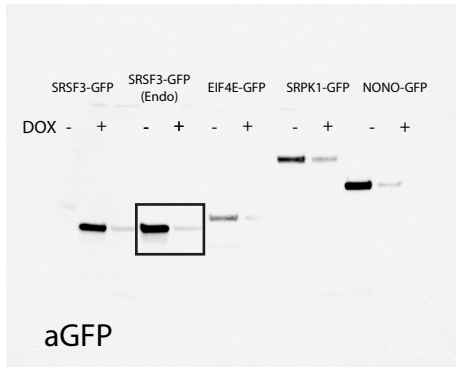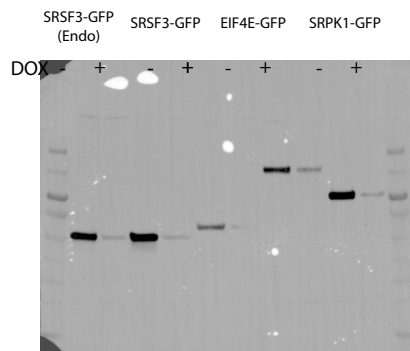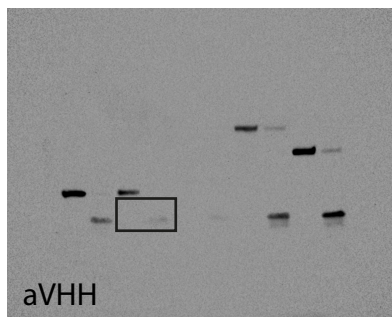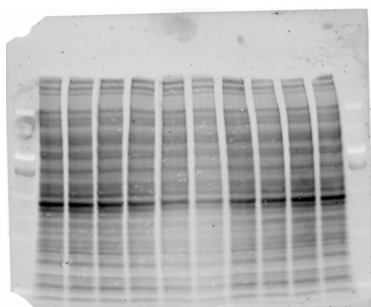

Stainfree

P19 SRSF5 Endo

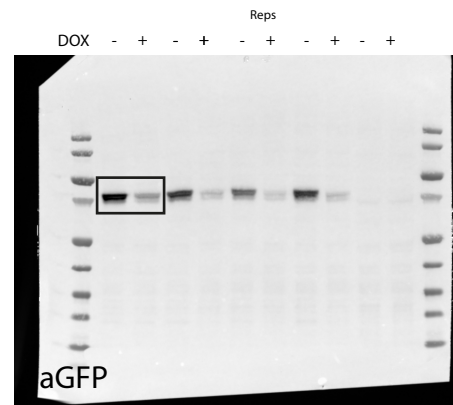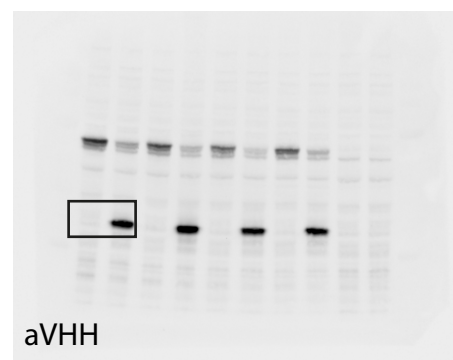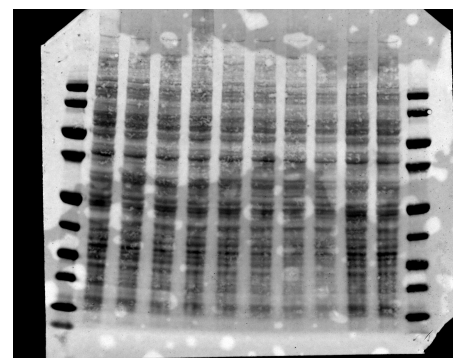

Stainfree

Fig.S4B

P19 endo-GFP (Timeline)

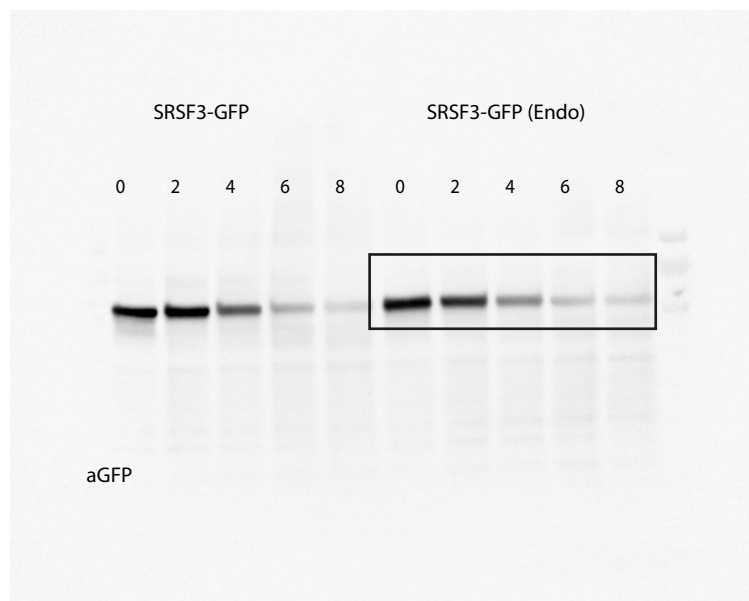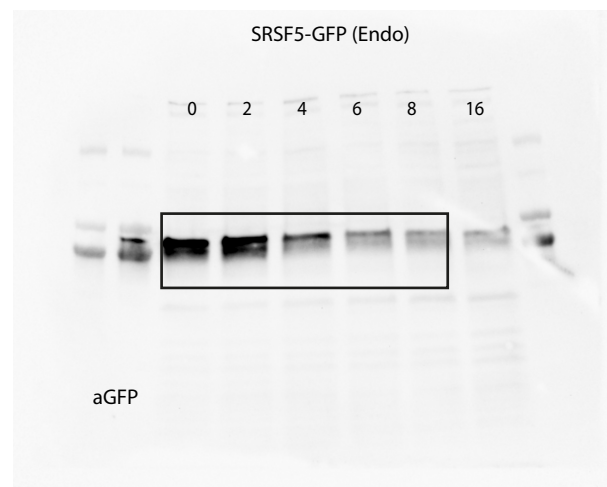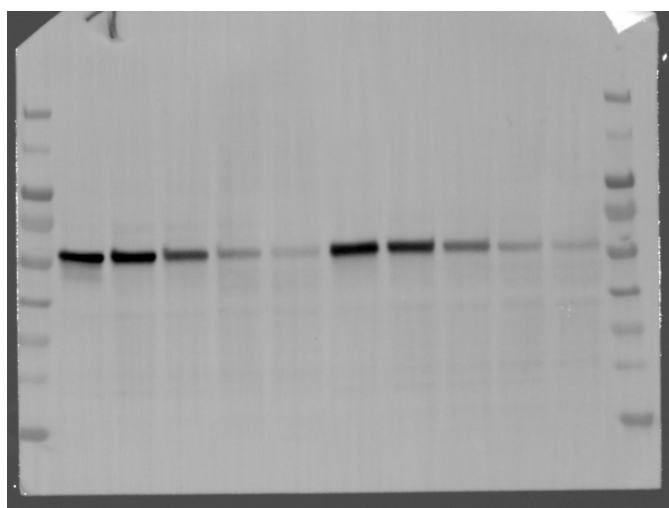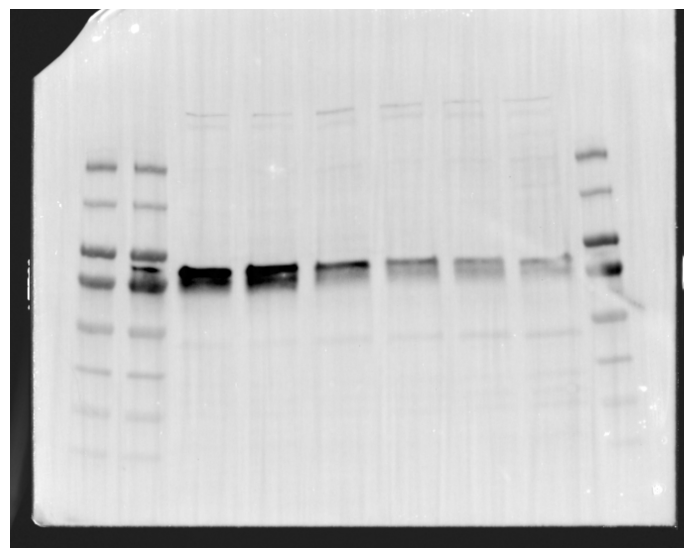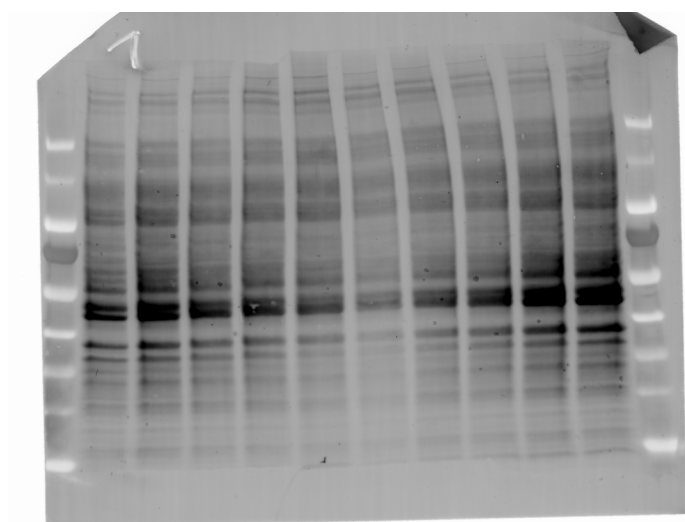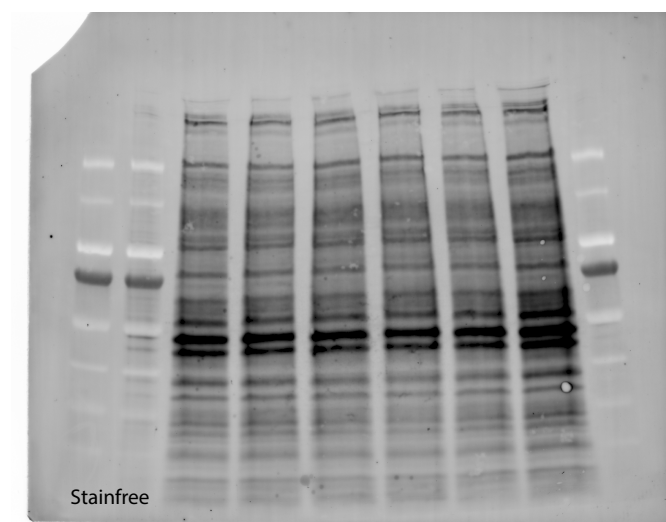

Fig.S4C

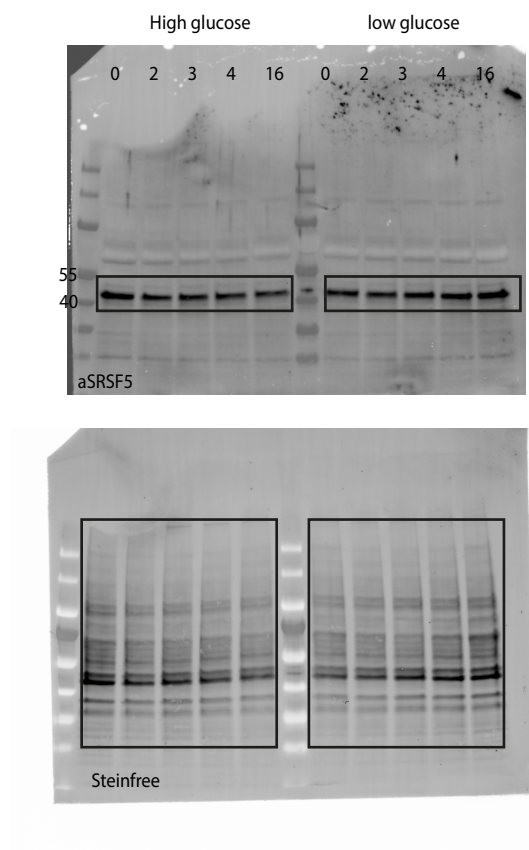

Flg.S4D

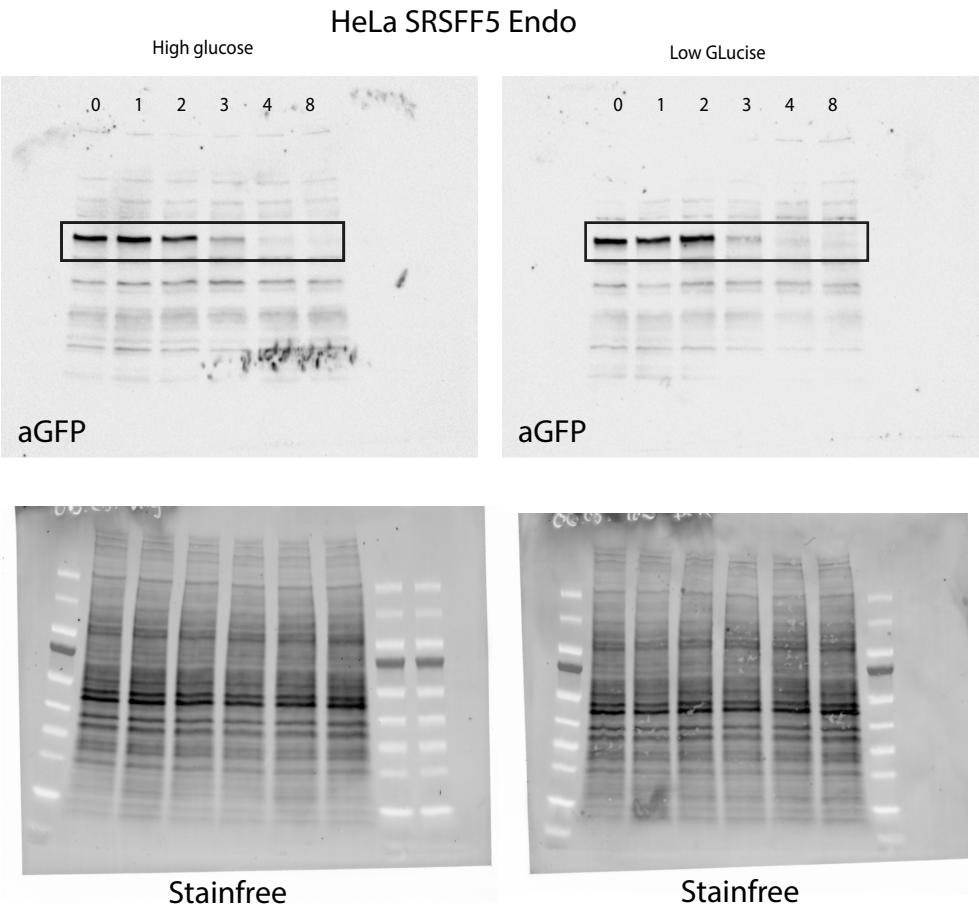

Supplement: SourceData FS4 — is the source file for Fig. S4. [file JCB_202304030_SourceDataFS4.pdf]

Fig.S5A

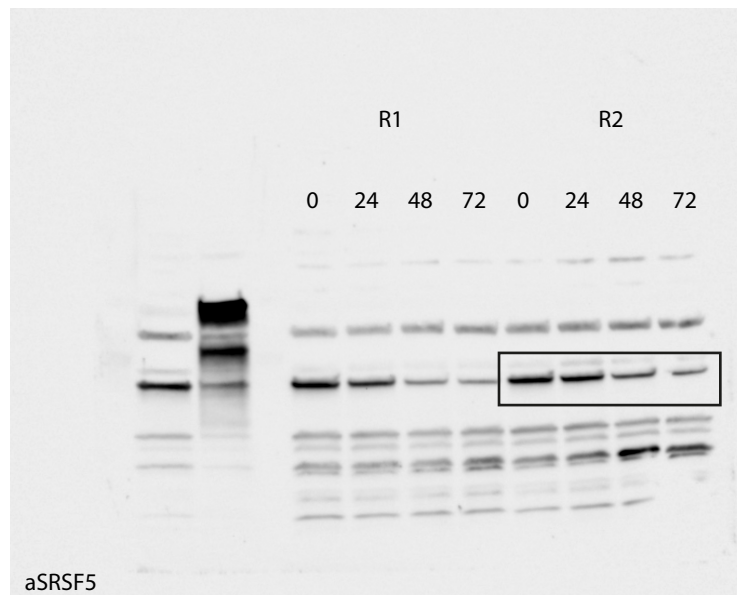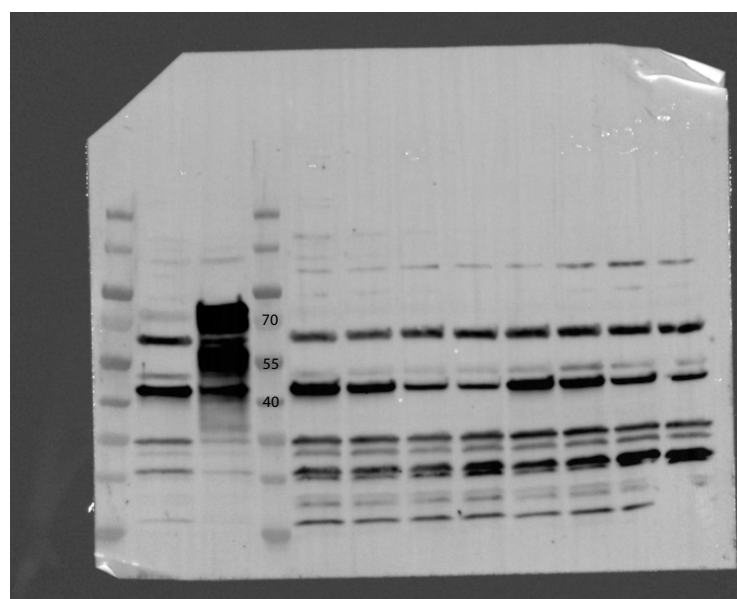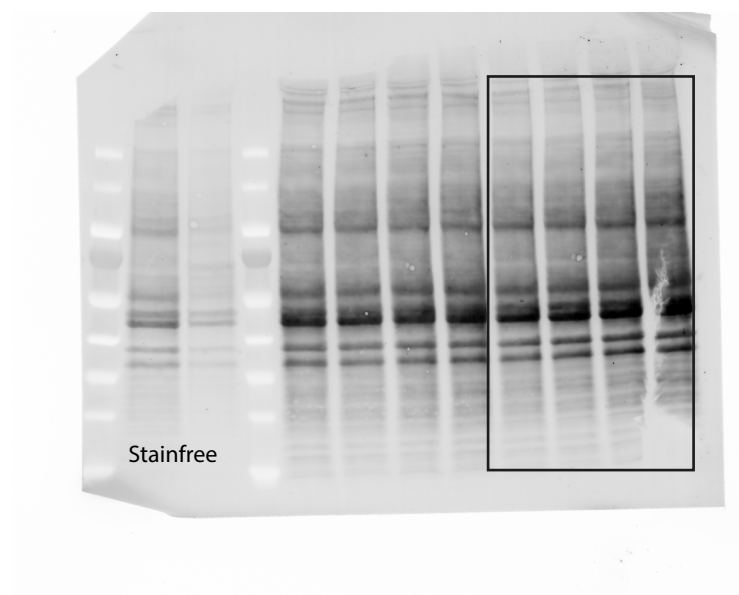

Fig.S5B

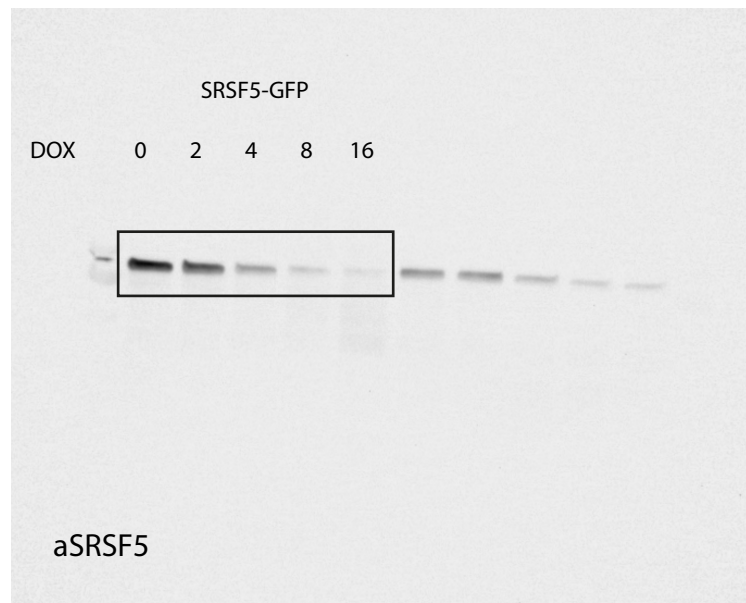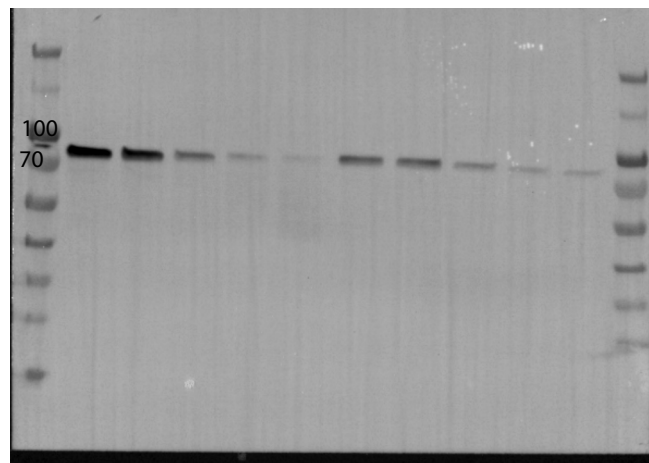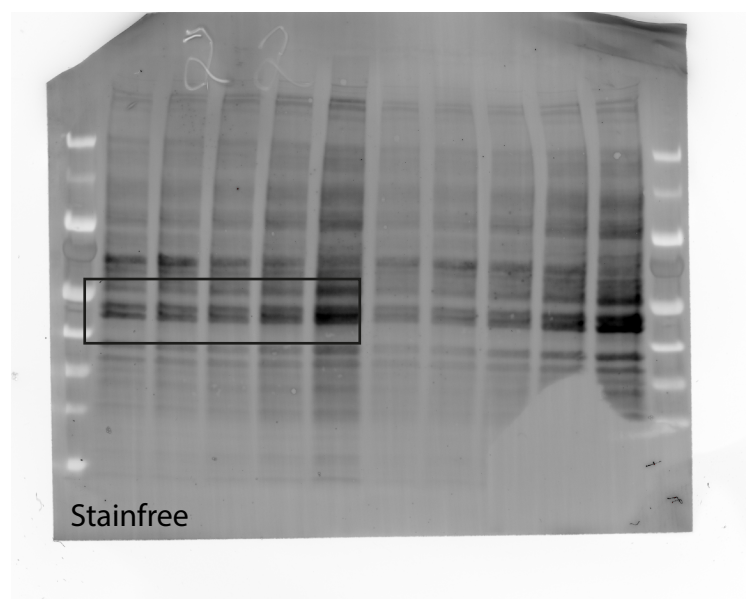

Supplement: SourceData FS5 — is the source file for Fig. S5. [file JCB_202304030_SourceDataFS5.pdf]
