# Supplementary material for: hGRAD: A versatile “one-fits-all” system to acutely deplete RNA binding proteins from condensates
Source: J Cell Biol. 2023 Dec 18;223(2):e202304030. doi: 10.1083/jcb.202304030 (PMC10726014; doi:10.1083/jcb.202304030)

FIG.4A

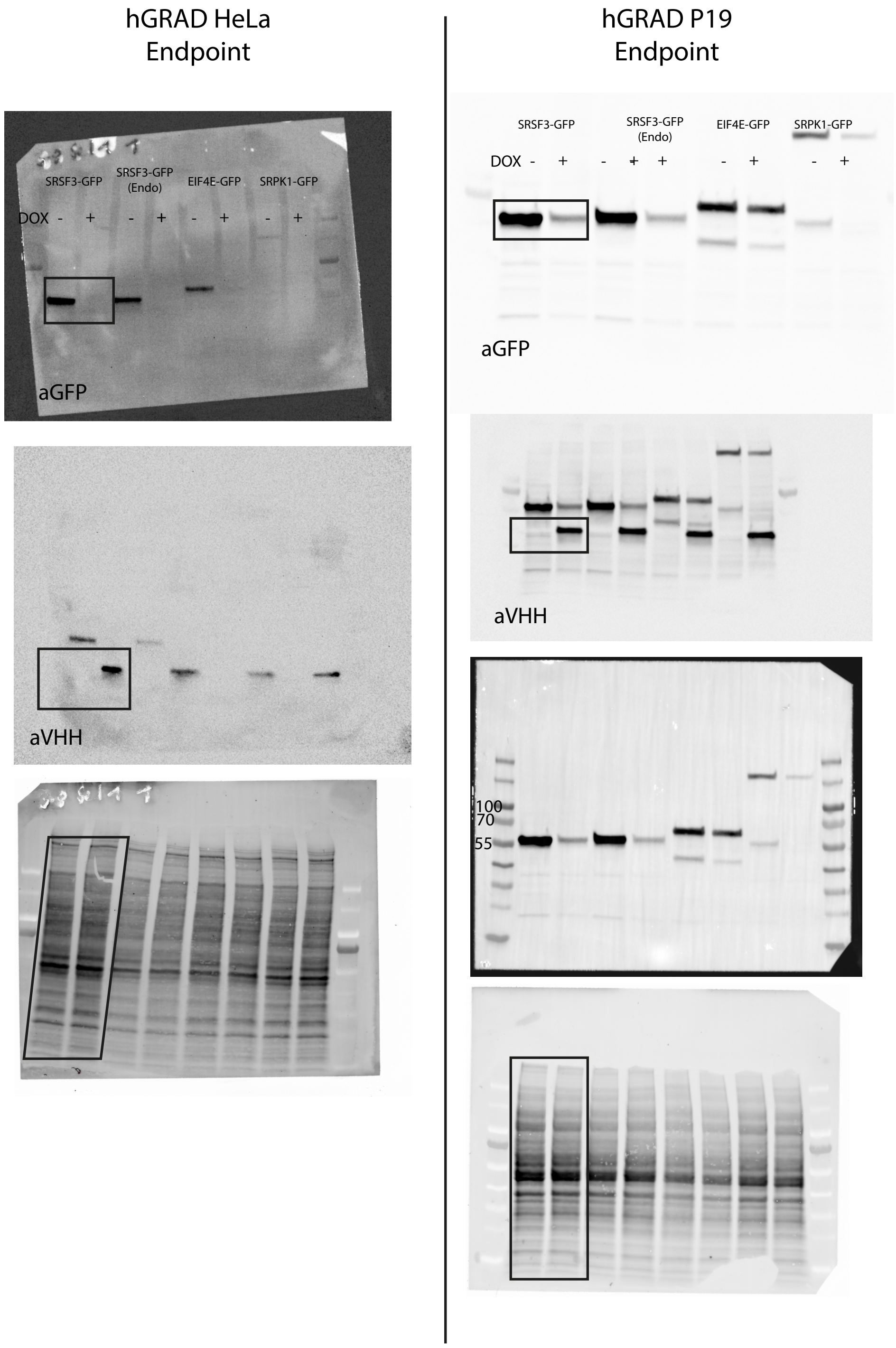
hGRAD HeLa
Endpoint
SRSF3-GFP
SRSF3-GFP
(Endo)
EIF4E-GFP
SRPK1-GFP
DOX - + - + - + - +
aGFP
aVHH
hGRAD P19
Endpoint
SRSF3-GFP
SRSF3-GFP
(Endo)
EIF4E-GFP
SRPK1-GFP
DOX - + - + + - + - +
aGFP
aVHH
100
70
55

FIG.4C

P19 hGRAD + BACs
Endpoint

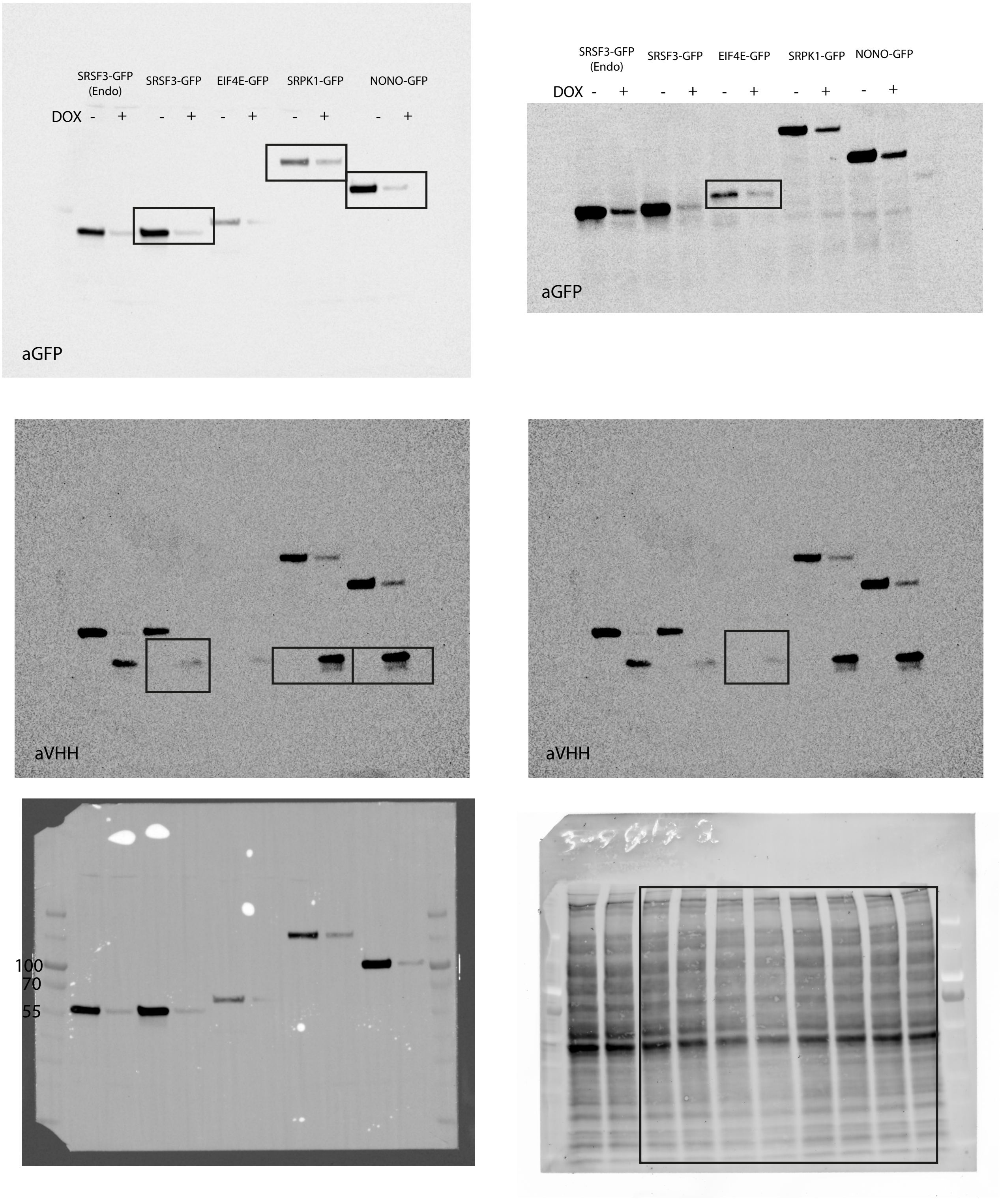

Stainfree

Fig4D

P19 hGRAD + BACs Timeline

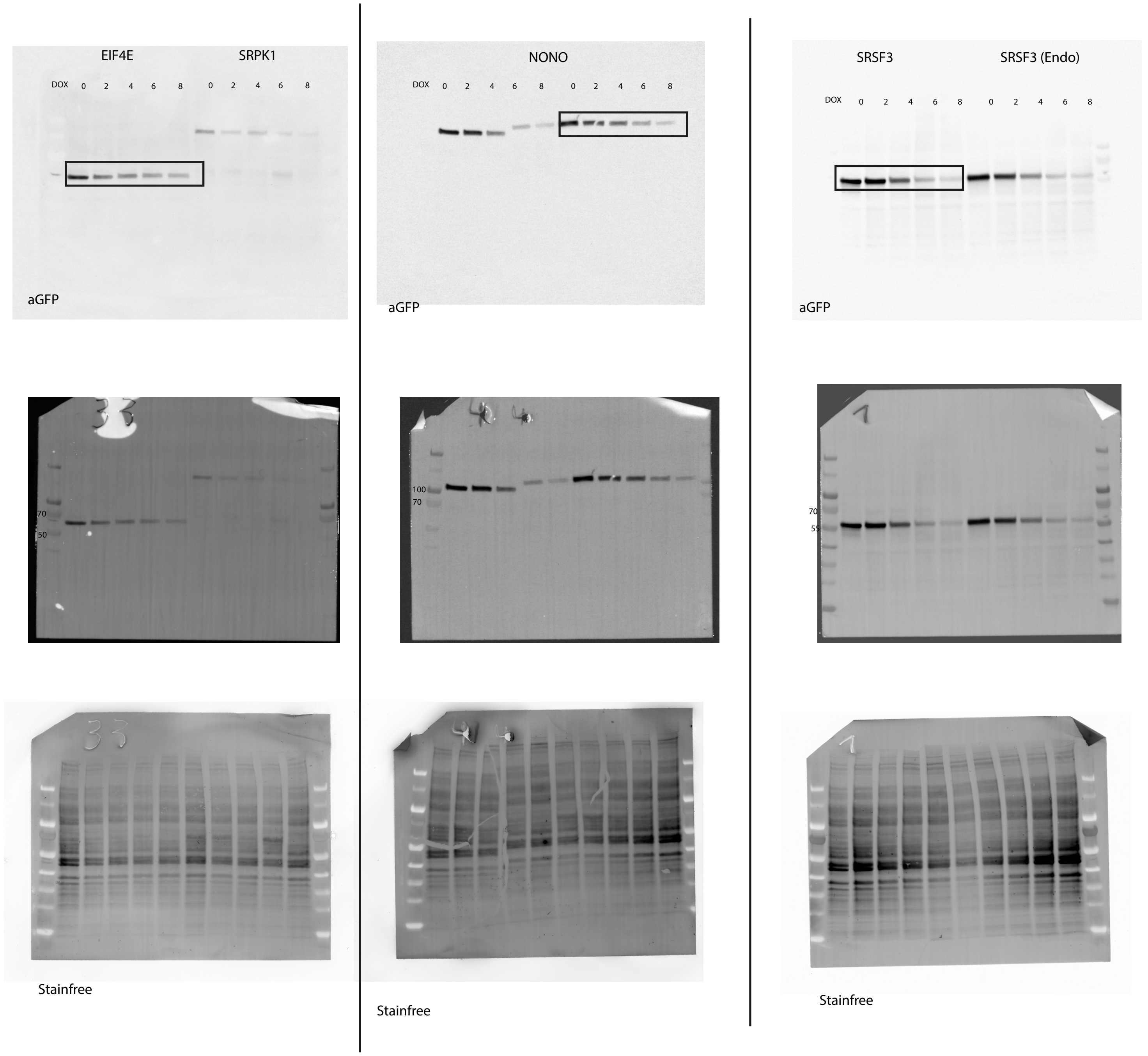

Supplement: SourceData F4 — is the source file for Fig. 4. [file JCB_202304030_SourceDataF4.pdf]
